# Detection of *Enterococcus avium* in a case of urinary tract infection and haematuria

**DOI:** 10.1099/acmi.0.000349

**Published:** 2022-05-16

**Authors:** Abdelrhman Abo-Zed, Shaymaa Hegazy, Tung Phan

**Affiliations:** ^1^​ Department of Medicine, University of Pittsburgh, Pittsburgh, Pennsylvania, USA; ^2^​ Department of Pathology, University of Pittsburgh, Pittsburgh, Pennsylvania, USA

**Keywords:** haematuria, urinary tract infection, *Enterococcus avium*, VITEK 2

## Abstract

Enterococci have been recognized as major pathogens causing nosocomial and community-acquired infections. The emergence of antimicrobial-resistant enterococci is one of the major public health challenges worldwide. While many enterococcal species have been identified, *Enterococcus avium* is rarely detected in humans. Here we present an interesting case of urinary tract infection and haematuria involving *E. avium* in a 72-year-old patient. The patient underwent antibiotic therapy and surgical procedures with excellent improvement. This case report highlights the important role of *E. avium* in clinical settings.

## Introduction

Enterococci are Gram-positive, catalase-negative and non-spore-forming bacteria that usually inhabit the alimentary tract of humans and animals [1, 2]. Enterococci are known to be important pathogens in humans, and their ability to inherit or acquire antibiotic resistance determinants is a global public health issue, causing significant morbidity and mortality [3]. Vancomycin resistance in enterococci was first reported in the UK in 1986, and the rapid increase of vancomycin-resistant enterococci (VRE) raised alarms in many countries [4, 5]. VRE infections are associated with increased mortality, longer length of stay, increased risk of discharge to a long-term care facility or readmission and higher costs [6]. Severe enterococcal infection-associated morbidity and mortality remain as high as 20–40 %, despite advances in antimicrobial therapy over the past few decades [7]. Enterococci are also common nosocomial pathogens, being responsible for approximately 14 % of hospital-acquired infections in the USA [8]. To date, approximately 58 different enterococcal species have been identified. *Enterococcus faecalis* and *Enterococcus faecium* are the most common, and they cause a variety of infections in humans, including bacteraemia, endocarditis, urinary tract infections (UTIs), prostatitis, intra-abdominal infection, cellulitis and wound infection [9, 10]. *E. avium* is also a member of the genus *Enterococcus *, and this species is mostly found in birds. *E. avium* is rarely a cause of infection in humans. In this study, we present an interesting case in which *E. avium* was isolated from urine collected from a patient showing UTI symptoms and haematuria.

## Case presentation

A 72-year-old patient with a history of type 2 diabetes, hypertension, endometrial cancer and atrial fibrillation initially had symptoms of urinary frequency and urgency, as well as dysuria and haematuria.

**Fig. 1.**
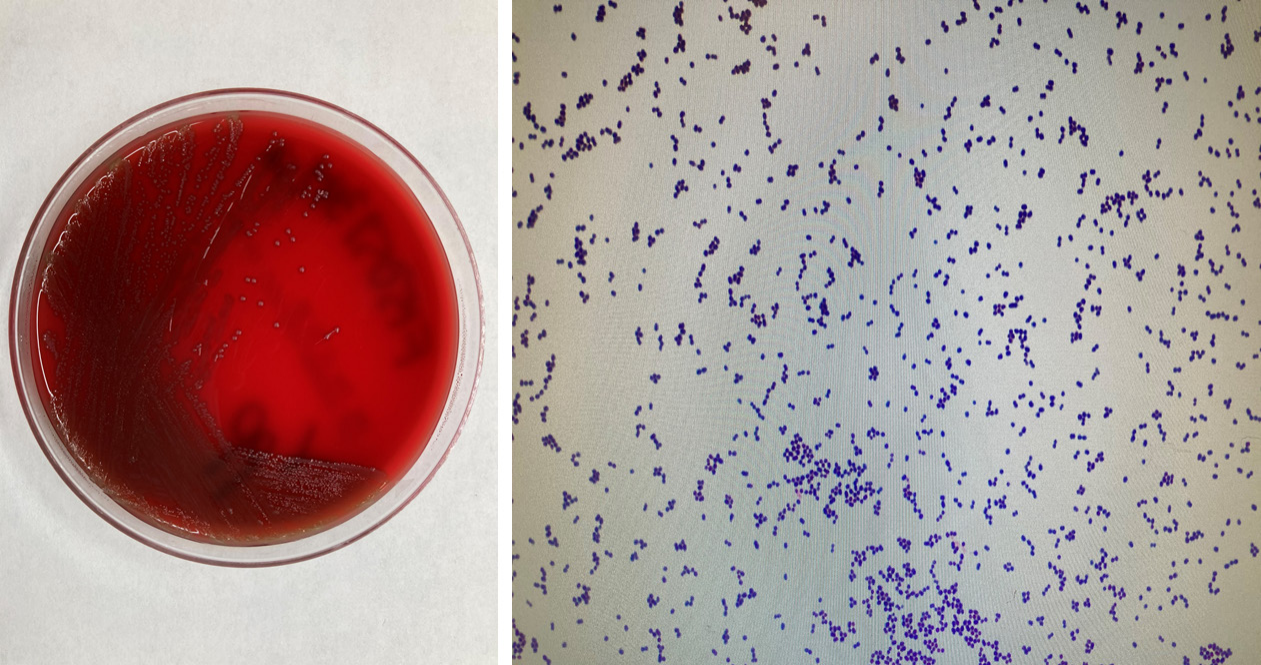
Small, grey, non-haemolytic colonies of *Enterococcus avium* were observed on sheep blood agar, and microscopic examination of a Gram-stained smear revealed small Gram-positive cocci at 1000× magnification.

The patient went to a primary care physician and was treated with a 1 week course of cefuroxime for uncomplicated cystitis. A urine specimen was collected and submitted to our clinical microbiology laboratory for bacterial culture. After 24 h of incubation at 35 °C in 5 % CO_2_, growth was observed on nonselective blood and chocolate agar plates. Small, grey, non-haemolytic colonies grew on the selective Columbia CNA agar plate. Gram-positive cocci were seen on the microscopic examination of a Gram-stained smear, as shown in Fig. 1. The isolate was identified as *E. avium* by the VITEK 2 system with an excellent confidence score of 99 %. Other bright reddish-pink colonies were also seen to grow on the selective MacConkey agar plate. These colonies were Gram-negative bacilli, and were identified as *Escherichia coli *.

**Fig. 2.**
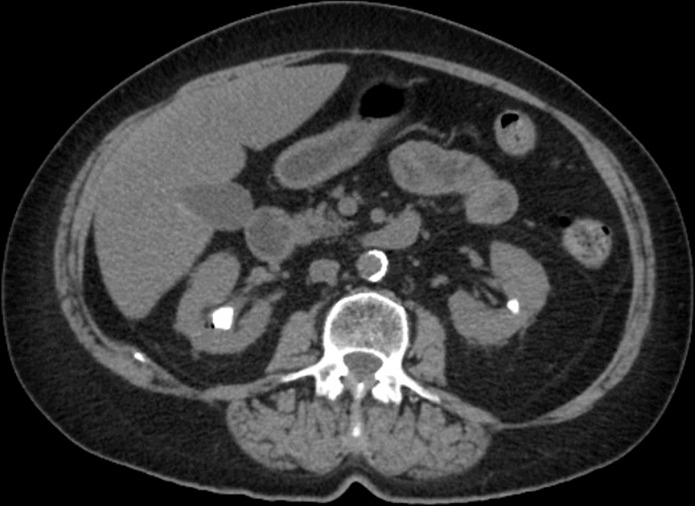
Abdominal and pelvic CT. The cut showed a 2.8 cm staghorn stone in the upper pole of the right kidney and a 8 mm non-obstructing stone in the interpolar region of the left kidney.

After completing the full course of antibiotics as prescribed, the UTI symptoms improved significantly; however, the painless haematuria persisted. The patient reported that the bloody urine was visible in the toilet bowl, and there was also a small amount on the toilet paper as the patient wiped. The patient denied any significant bleeding into the undergarments between voids. A newly collected urine specimen was submitted for bacterial culture. There was no growth on the agar after 48 h of incubation, and the urine culture was considered negative. Urinary cytology was negative for high-grade urothelial carcinoma. Computed tomography of the abdomen and pelvis showed a 2.8 cm staghorn stone in the upper pole of the right kidney and a 8 mm nonobstructing stone in the interpolar region of the left kidney (Fig. 2). No focal renal lesions were identified. Ureteral stones or hydronephrosis were not noted. However, there was diffuse wall thickening and oedema of the bladder. Cystoscopy revealed a bleeding lesion just to the right of the bladder neck that was mildly ulcerated. The lesion (approximately 0.5 cm) was resected, and the entire base of the resection site was cauterized. The patient also underwent the percutaneous nephrolithotomy to remove the staghorn stone in the right kidney. The patient was eventually discharged and did well on the follow-up visit.

## Discussion

*E. avium *, formerly ‘group Q streptococcus’, has rarely been known as a pathogen in humans. During the 12 years from 1997 to 2009, there were 53 patients with *E. avium* bacteraemia at a tertiary-care hospital in the Republic of Korea, and the mortality rate was 11.3 % [11]. Mohanty and co-authors found the first case of brain abscess due to *E. avium* in a 19-year-old man with chronic otitis media since childhood [12]. This bacterium has been isolated in abscess aspirates from different organs, such as the spleen and pancreas [13, 14]. Only a few other clinical diseases caused by *E. avium* have been reported, including endocarditis, osteomyelitis, peritoneal dialysis-associated peritonitis and cholecystitis [15–18]. However, UTI and haematuria associated with *E. avium* are very rare. Here we present an interesting case of UTI and haematuria involving *E. avium* in an adult patient. Since *E. avium* is very commonly found in birds, one of the risk factors for *E. avium* infection is contact with birds. There is no evidence that the *E. avium* infection in this patient was acquired through direct contact with infected birds or contaminated environments. Therefore, the source of infection could not be identified. The clinical significance of *E. avium* is questionable in causing UTI and haematuria in this clinical case, since *E. coli* was also isolated in the same urine specimen. *E. coli* has been documented to be the most prevalent agent of uncomplicated and complicated UTIs. It is important to point out that *E. avium* grew a high number of 50 000 colony-forming units (c.f.u.) ml^−1^ in the urine specimen, suggesting its causative role in this clinical case. Our keyword search (* E. avium* and UTI or haematuria or cystitis) in PubMed identified only two previously published studies [19, 20]. Ishihara *et al*. reported 24 *E. avium* strains isolated from Japanese patients with complicated UTIs between 1988 and 2000 [19]. In northern India, *E. avium* was isolated in two urine specimens collected from patients diagnosed with community-acquired UTIs [20]. In addition, *E. avium* was reported in blood and the *E. avium* bacteraemia-related mortality rate was 11.3 % [21]. This rare micro-organism was also found in cerebrospinal fluid and caused meningoencephalitis [22]. Taken together, we should consider *E. avium* to be a clinical pathogen associated with UTI and haematuria in this case. The association of *E. avium* with UTI and haematuria further extends the clinical spectrum of this rare pathogen.

## Conclusion

*E. avium* has emerged as a pathogen causing a variety of human infections. The detection of *E. avium* in urine collected from this case and in sterile sites such as blood or cerebrospinal fluid from the previous reports highlights the important role in the pathogenicity of *E. avium* in humans. To the best of our knowledge, our case study is the first report showing the possible association of *E. avium* and haematuria. Although *E. avium* is thought to have low virulence, a high mortality rate in patients with bacteraemia, especially in those with severe underlying conditions, has been reported. Therefore, accurate diagnosis and early treatment are essential to achieve a successful outcome.
